# Detection of *Leptospira* in cane toads (*Rhinella jimi*) from urban and rural Paraíba, Brazil

**DOI:** 10.1002/vetr.70228

**Published:** 2026-01-04

**Authors:** Karla N. de Souza Rocha, Aline de Lima Peixoto, Lucila S. de Medeiros Pereira, Debora Ferreira Cardoso, Maria L. C. Rodrigues Silva, Sérgio S. de Azevedo, Juliana P. F. de Oliveira, Clebert J. Alves, Mozar Alves Fonseca, Severino S. dos Santos Higino

**Affiliations:** ^1^ Graduate Program in Animal Science and Health Federal University of Campina Grande Patos Brazil; ^2^ Federal Institute of Paraíba (IFPB) Patos Brazil; ^3^ Federal University of Sergipe Nova Esperança Brazil; ^4^ Department of Animal and Range Sciences, Clayton Livestock Research Center New Mexico State University Clayton New Mexico USA

**Keywords:** amphibians, herpetofauna, leptospirosis, One Health, reservoirs

## Abstract

**Background:**

Leptospirosis is a significant zoonosis in tropical regions, where poor sanitation and favourable climate aid its spread. Synanthropic animals such as the cane toad (*Rhinella jimi*), which share environments with both people and wild and domestic animals, may harbour *Leptospira* and contribute to urban and rural transmission cycles.

**Methods:**

This study evaluated the serological and molecular presence of *Leptospira* spp. in *R. jimi* collected from urban and rural areas in Brazil's semi‐arid region. Twenty‐five toads were captured, euthanased and sampled. Blood was analysed using the microscopic agglutination test, and urine and kidney tissues were tested via polymerase chain reaction (PCR) targeting the LipL32 gene.

**Results:**

Six toads (24%) were seropositive for *Leptospira*, with the identified serogroups being Australis (*n* = 3), Icterohaemorrhagiae (*n* = 2) and Pomona (*n* = 1). PCR detected *Leptospira* DNA in 11 animals (44%), mainly from urban locations.

**Limitations:**

The study's small sample size, although ethically justified, represents a limitation for broader epidemiological inferences. Furthermore, due to the lack of complete data on the animals’ prior movements, the relationship between infection and geographical origin could not be fully determined.

**Conclusion:**

The presence of antibodies and leptospiral DNA indicates that *R. jimi* may act as a non‐conventional host in *Leptospira* transmission, posing a potential public health risk.

## INTRODUCTION

Leptospirosis is a globally significant zoonotic disease that is transmitted to people through contact with contaminated water or direct exposure to the urine of infected animals.^1^ There are an estimated 1.03 million human cases and 58,900 disease‐related deaths annually, with Oceania exhibiting the highest morbidity (150.68 cases per 100,000 inhabitants per year) and mortality (9.61 deaths per 100,000 inhabitants per year).^2^ Continuous alterations in ecosystems and wildlife habitats, combined with increased movement of animals into urban and peri‐urban areas, have intensified interactions between wild and domestic species, amplifying the epidemiological cycle of the disease.^3^

Environmental and socioeconomic factors, such as inadequate sanitation, overcrowding in areas with poor infrastructure and tropical climatic conditions, facilitate the persistence of *Leptospira* spp. in the environment and contribute to seasonal increases in human infections.^4^ Leptospirosis is widely recognised as a poverty‐associated disease and is included among neglected tropical diseases, with growing attention even in developed countries due to climate change, as well as increased international travel and trade with endemic regions.^5^

The genus *Leptospira* comprises 66 species and over 300 serovars. Advances in molecular diagnostics, increased surveillance, broader ecological monitoring and environmental changes, including urbanisation, habitat fragmentation and climate variability, have expanded the host range of these pathogens.^6^ Genomic studies indicate gene gain and loss and adaptive evolution, particularly regarding virulence and host specificity, although direct links between genomic changes and host shifts remain limited.^6^, ^7^ Many serovars persistently colonise the renal tubules of maintenance hosts and are shed in urine throughout the host's lifetime.^8^ Additionally, biofilm formation enhances environmental persistence in soil and water, even under nutrient‐limited conditions.^8^, ^9^, ^10^

Mammals represent the primary hosts of *Leptospira*,^11^ including dogs,^12^ cattle,^13^, ^14^ horses,^15^ cats,^16^ pigs^17^ and small ruminants.^18^ However, infections have also been reported in synanthropic^1^ and wild animals,^19^, ^20^ significantly expanding the known host range. A review conducted by Cilia et al.^3^ documented 34 studies, published between 1960 and 2020, reporting *Leptospira* in non‐adapted species, which, although not typical reservoirs, may contribute to the transmission and maintenance of the pathogen in nature.

In recent decades, the host spectrum has further expanded to include atypical species such as birds (gulls, pigeons, ibises and owls),^21^ reptiles^22^ and fish.^23^ While most *Leptospira* isolations have occurred within the orders Carnivora, Didelphimorphia and Rodentia, additional reports exist in Cetacea, Cingulata, Afrosoricida, Chiroptera, Primates, Reptilia and Amphibia.^3^, ^24^

Evidence of pathogenic *Leptospira* has also been described in amphibians, including cane toads (*Bufo marinus*) and whistling frogs (*Eleutherodactylus johnstonei*) in Barbados, which showed reactivity to serovars Bim and Bajan (serogroup Australis, Leptospira *interrogans*), although no clinical disease was observed.^25^ Infection in these organisms may occur through exposure to contaminated water, with cutaneous absorption of the pathogen, storage in the urinary bladder, and possible reabsorption during dehydration.^22^

Amphibians often inhabit anthropogenic environments, exploiting humid locations shared with rodents, the primary reservoirs of *Leptospira*,^19^ and areas with artificial lighting that attract insect prey, their main food source. A particularly relevant species is the cane toad (*Rhinella jimi*, formerly *Bufo jimi*), a member of the *Rhinella marina* complex,^26^ which is widely distributed in urban and semi‐arid areas of northeastern Brazil.^27^, ^28^ This species is strongly associated with aquatic ecosystems essential for reproduction and cutaneous respiration, inhabiting sites near streams, rivers, ponds and swamps—environments often linked to high *Leptospira* incidence.^29^, ^30^, ^31^ Its predators include hedgehogs, rodents, mustelids and domestic cats.^28^, ^29^

Although mammals, particularly *Rattus norvegicus*, remain the main reservoirs and sources of human infection,^30^, ^31^ the epidemiological role of amphibians and reptiles (herpetofauna) in the maintenance and dissemination of *Leptospira* remains poorly understood. Recent studies have detected *Leptospira* DNA or antibodies in turtles, snakes and frogs, suggesting incidental infections or environmental persistence of the bacterium.^32^, ^33^, ^34^, ^35^ Zoonotic transmission involving herpetofauna has also been reported, especially in aquatic turtles,^36^, ^37^ while terrestrial species, such as snakes, may contribute to soil contamination in forest habitats, facilitating transmission to small mammals and other susceptible hosts.^38^

Moreover, molecular studies reinforce the circulation of *L. interrogans* in Brazil's semi‐arid biome. Silva et al.^39^ sequenced *L. interrogans* strains from ovine samples in the state of Paraíba, while Silva et al.^40^ isolated *Leptospira* spp. from *Cavia aperea*, *Euphractus sexcinctus* and *Cerdocyon thous* in the semi‐arid region of Ceará. These findings demonstrate the presence of the pathogen across a wide range of animal species, likely supported by genetic traits that enhance environmental resilience and biofilm‐forming capacity, thus promoting bacterial survival and dissemination even during dry periods.^41^, ^42^

Despite limited current knowledge, accumulating evidence supports the potential role of amphibians as non‐mammalian hosts in the ecology of leptospirosis, underscoring the need for expanded ecological surveillance that includes unconventional species. Given the widespread occurrence of *R. jimi* in anthropised areas of the semi‐arid Caatinga biome and the limited understanding of its contribution to the transmission of pathogenic *Leptospira*, this study aimed to assess the serological and molecular occurrence of *Leptospira* spp. in *R. jimi* collected under semi‐arid conditions.

## MATERIALS AND METHODS

### Study location

A cross‐sectional analytical observational study was conducted in the southern zone of the municipality of Patos (latitude: 07°01′28″S; longitude: 37°16′48″W), located in the state of Paraíba, Brazil. The study covered the neighborhoods of Monte Castelo, Jatobá, Santa Cecília, Nova Conquista, Alto da Tubiba and nearby rural communities surrounding the Jatobá.

The municipality encompasses an area of 472.892 km^2^, distributed across one district, 23 neighbourhoods, two localities and three rural zones. The estimated population is 103,165 inhabitants, with a demographic density of 218.16 people per km^2^.^43^, ^44^ Approximately 41.7% of the population lives on a monthly per capita income of up to half the minimum wage, thus classified as low‐income families by the Brazilian Ministry of Social Development and the Fight Against Hunger. These families are predominantly concentrated in the peripheral areas covered by this study.^44^, ^45^ Additionally, 86.64% of the population resides in areas lacking basic sanitation infrastructure, 8.06% do not have access to public waste collection services, and 2% of households are vulnerable to flooding, considering the region's typical annual rainfall concentrated between January and April.^45^

Patos city is located in a hot and dry semi‐arid region, characterised by typical Caatinga vegetation and a fauna threatened by urban expansion and uncontrolled extractive activities, such as charcoal production and wood harvesting for industrial use, leading to desertification in certain areas.^44^ One of the species present in the region's fauna is *R. jimi*, commonly known as the cane toad, found in both urban and rural environments of the city, and selected as the target population for this study.

### Ethical considerations and study population

Following the identification of the target species and approval by the Brazilian Institute of Environment and Renewable Natural Resources (BIERNR)/Chico Mendes Institute for Biodiversity Conservation (ICMBio—Brazil) under permit no. 70478‐1 and Animal Use Ethics Committee of the Federal University of Campina Grande (FUCG) under opinion no. 089/2019, medium‐sized juvenile toads were captured using simple manual collection techniques without the need for traps, given their ease of handling. The final sample consisted of 25 individuals, 16 of which were captured in urban areas and nine in rural areas (Table 1). Each toad was then assigned an identification code starting with atypical host (AH), followed by a numerical designation (AH1‒AH25), corresponding to the order of inclusion in the study.

The animals were then transported to the Laboratory of Transmissible Diseases (LTD) at the Center for Health and Rural Technology (FUCG), where euthanasia was performed prior to sample collection. Blood samples were obtained for serological testing, and urine and kidney samples were collected for molecular diagnosis using polymerase chain reaction (PCR).

Euthanasia was carried out in accordance with the guidelines of Normative Resolution no. 37, dated 15 February 2018, issued by the Brazilian National Council for the Control of Animal Experimentation, which outlines the euthanasia procedures by taxonomic group. Specifically, item 9.7—amphibians, fish and reptiles—was followed. Procedures were conducted in a clean, noise‐free environment at the LTD, isolated from other animals and under low light conditions, in order to respect the species’ specific physiological and behavioural characteristics and to minimise pain and distress before and during the procedure.^46^

Given the species’ sensitivity, ability to secrete mucus and toxic substances, and the high permeability of its skin, the toads were first anaesthetised by topical application of 0.5 mg/mL lidocaine to induce rapid loss of consciousness and central nervous system depression. This was followed by potassium chloride injection and sensitivity testing to confirm cardiopulmonary arrest, asystole and irreversible loss of brain function.

As the total population size of the biological species under study was unknown, each sampled individual was considered a significant fraction of a finite, unmatched universe. Therefore, the sample size calculation applied a finite population correction factor, thereby reducing the minimum number of animals required.^47^ The minimum sample size was obtained using the following formula:

$$n=\left(Sa^{2}+Sb^{2}\right)\times {\left[\frac{\left(Z\alpha /2\right)+Z\beta }{d}\right]}^{2}$$

where *n* is the sample size (for each subgroup); *Zα*/2 is the error value *α*, usually: 1.96 (5%); *Zβ* is the error value *β*, usually: 0.84 (20%); *d* is the minimum difference between the means; and Sa and Sb are the standard deviations of the variable in each group.

### Collection and processing of blood, urine and kidney tissue samples

Blood samples (3.0 mL) were collected via cardiac puncture using individual disposable needles and syringes. After collection, each sample was transferred to an 8.5 mL non‐anticoagulant tube containing a clot activator, properly labelled, and centrifuged at 1512 × *g* for 10 minutes. The resulting serum was extracted in duplicate, transferred to 2 mL microtubes, and stored at ‒20°C until serological testing.

Molecular diagnostics were performed using approximately 300 µL of urine and 500 ng (0.5 µg) of kidney tissue from each toad. After appropriate labelling and storage in DNase‐ and RNase‐free microtubes, samples were sent to the Laboratory of Molecular Biology of the Semi‐Arid at the FUCG.

### Serological testing

Microscopic agglutination testing (MAT) was conducted to diagnose *Leptospira* spp. infection,^48^ using a panel of 23 pathogenic and two saprophytic serovars. These included Australis, Autumnalis, Ballum, Bratislava, Castellonis, Canicola, Celledoni, Copenhageni, Cynopteri, Djasiman, Grippotyphosa, Hebdomadis, Icterohaemorrhagiae, Louisiana, Mini, Panama, Pomona, Pyrogenes, Hardjoprajitno, Wolffi, Shermani, Tarassovi and Guaricurus. All antigens (Table 2) were kindly provided by the Veterinary Bacteriology Laboratory at the Fluminense Federal University, Niteroi, Rio de Janeiro, Brazil, sourced from the Pasteur Institute (France), from the Oswaldo Cruz Foundation and World Health Organization, and maintained in Ellinghausen‐McCullough‐Johnson‐Harris (EMJH) liquid medium. Antigen cultures were examined under dark‐field microscopy (100×) prior to testing to confirm purity and the absence of auto‐agglutination.

**TABLE 1:** Anatomical and spatial characteristics of the 25 cane toads captured in urban and rural areas of Patos municipality, Paraíba state, Brazil.

Sample	Location	Area	Collection date	SVL length (mm)	Weight (g)	Nuptial pad	Sex	Skin lesions
AH1	Santa Cecília district	Urban zone	04/05/2020	110	320	Yes	Male	No
AH2	Santa Cecília district	Urban zone	04/05/2020	108	290	Yes	Male	No
AH3	Santa Cecília district	Urban zone	04/05/2020	105	280	Yes	Male	No
AH4	Santa Cecília district	Urban zone	04/07/2020	140	620	No	Female	No
AH5	Santa Cecília district	Urban zone	04/07/2020	115	310	Yes	Male	No
AH6	Santa Cecília district	Urban zone	04/07/2020	135	590	No	Female	No
AH7	Santa Cecília district	Urban zone	04/07/2020	112	300	Yes	Male	No
AH8	FUCG farm	Rural zone	04/13/2020	145	650	No	Female	No
AH9	FUCG farm	Rural zone	04/13/2020	130	540	No	Female	No
AH10	FUCG farm	Rural zone	04/13/2020	138	600	No	Female	No
AH11	Alto da Tubiba district	Urban zone	04/13/2020	113	310	Yes	Male	No
AH12	Alto da Tubiba district	Urban zone	04/13/2020	142	620	No	Female	No
AH13	FUCG farm	Rural zone	04/16/2020	118	330	Yes	Male	No
AH14	FUCG farm	Rural zone	04/16/2020	139	610	No	Female	No
AH15	FUCG farm	Rural zone	04/16/2020	109	295	Yes	Male	No
AH16	Monte Castelo district	Urban zone	04/19/2020	144	640	No	Female	No
AH17	Monte Castelo district	Urban zone	04/19/2020	111	305	Yes	Male	No
AH18	Jatobá district	Urban zone	04/19/2020	140	625	No	Female	No
AH19	Jatobá district	Urban zone	04/19/2020	115	315	Yes	Male	No
AH20	Jatobá district	Urban zone	04/21/2020	110	290	Yes	Male	No
AH21	Nova Conquista district	Urban zone	04/22/2020	137	600	No	Female	No
AH22	Nova Conquista district	Urban zone	04/22/2020	143	635	No	Female	No
AH23	FUCG farm	Rural zone	05/13/2020	138	500	No	Female	No
AH24	FUCG farm	Rural zone	05/13/2020	139	610	No	Female	No
AH25	FUCG farm	Rural zone	05/13/2020	116	320	Yes	Male	No

Abbreviations: FUCG, Federal University of Campina Grande; SLV, snout‐vent length.

**TABLE 2:** Bacterial culture collection of veterinary interest used in the microscopic agglutination test at the Laboratory of Transmissible Diseases, Federal University of Campina Grande.

Species	Serogroup	Serovar	Strain	Source institution
*Leptospira interrogans*	Pyrogenes	Pyrogenes	Salinen	Pasteur Institute
*L. interrogans*	Pomona	Pomona	Pomona	Pasteur Institute
*Leptospira kirschneri*	Grippotyphosa	Grippotyphosa	Moskva V	Pasteur Institute
*L. interrogans*	Canicola	Canicola	Hond Utrecht IV	Pasteur Institute
*L. interrogans*	Australis	Australis	Ballico	Pasteur Institute
*L. interrogans*	Autumnalis	Autumnalis	Akiyami A	Pasteur Institute
*L. interrogans*	Icterohaemorrhagiae	Icterohaemorrhagiae	Verdun	Pasteur Institute
*Leptospira noguchi*	Lousiana	Lousiana	Luc 1945	Pasteur Institute
*L. interrogans*	Djasiman	Djasiman	Djasiman	Pasteur Institute
*Leptospira borgpetersenii*	Ballum	Castellonis	Castellon 3	Pasteur Institute
*L. noguchi*	Panama	Panama	CZ 214K	Pasteur Institute
*L. interrogans*	Australis	Bratislava	Jez Bratislava	Oswaldo Cruz Foundation
*Leptospira santarosai*	Shermani	Shermani	1342K	Pasteur Institute
*L. interrogans*	Icterohaemorrhagiae	Copenhageni	M20	Oswaldo Cruz Foundation
*L. borgpetersenii*	Ballum	Ballum	Mus 127	Oswaldo Cruz Foundation
*L. borgpetersenii*	Tarassovi	Tarassovi	Perepelitsin	Instituto Pasteur
*L. interrogans*	Sejroe	Wolffi	3705	Instituto Pasteur
*L. kirschneri*	Cynopteri	Cynopteri	3522C	Instituto Pasteur
*L. santarosai*	Sejroe	Guaricura	BOV G	Oswaldo Cruz Foundation
*L. interrogans*	Sejroe	Hardjoprajitno	Hardjoprajitno	World Health Organization
*L. interrogans*	Hebdomadis	Hebdomadis	Hebdomadis	Instituto Pasteur
*Leptospira weilli*	Celledoni	Celledoni	2011/01963	Instituto Pasteur
*L. borgpetersenii*	Mini	Mini	2008/01925	Instituto Pasteur

For MAT, sera were initially diluted 1:50 (0.1 mL serum + 4.9 mL Sorensen's phosphate‐buffered saline, pH 7.4). Then, 50 µL of diluted serum was placed in each well of a flat‐bottom 96‐well polystyrene microplate, followed by 50 µL of antigen. Each serum sample was tested against all 23 serovars, incubated at 28°C for 2 hours in a bacteriological incubator, and then analysed under optical microscopy to detect agglutination.

Samples that tested positive in the initial screening were subjected to serial twofold dilutions in duplicate (from 1:100 to 1:6400) to determine the final titres. The antibody titre was defined as the highest serum dilution that produced 50% or greater agglutination of live leptospires observed under dark‐field microscopy. The highest titre was considered representative of the serogroup showing the greatest serological reactivity.

Readings were performed using a ZEISS optical microscope (Oberkochen), equipped with a dry dark‐field condenser, 10×/0.20 objective, and 10× ocular lens (total magnification: 100×), allowing visualisation of agglutination patterns.

### Molecular analysis

DNA from kidney tissue and urine samples was extracted using the DNeasy Blood and Tissue Kit (Qiagen), following the manufacturer's instructions. Conventional PCR was performed using primers LipL32‐45F (5′‐AAGCATTACCGCTGGTG‐3′) and LipL32‐286R (5′‐GAACTCCCATTTCAGCGATT‐3′), as described by Stoddard et al.,^49^ targeting the *LipL32* gene, specific to pathogenic *Leptospira* strains.

Each PCR reaction mix (total volume of 50 µL) contained the following: 5 µL of extracted DNA, 32.8 µL of DEPC‐treated water, 5 µL of Taq DNA polymerase buffer, 1.0 µL of dNTPs, 3 µL of MgCl_2_ solution, 1.5 µL each of LipL32‐45F and LipL32‐286R primers, and 0.2 µL of Taq DNA polymerase.^50^ An *L. interrogans* strain (serogroup Pomona, serovar Kennewicki) was used as the positive control, and ultrapure water served as the negative control.

Reactions were carried out in a Gene Amp PCR System 9700 thermal cycler (Applied Biosystems). The cycling protocol consisted of an initial denaturation at 95°C for 5 minutes, followed by 35 cycles of denaturation at 94°C for 30 seconds, annealing at 53°C for 30 seconds and extension at 72°C for 1 minute. A final extension was performed at 72°C for 5 minutes. PCR products were analysed by electrophoresis in 2% agarose gels stained with Blue Green Loading Dye I, and DNA bands were visualised under UV light. The expected amplicon size was approximately 242 base pairs.

### Statistical analysis

The primary data were tabulated in Excel and analysed using descriptive statistics in SPSS software (IBM SPSS Statistics 20.0). Variables such as the proportion of positive animals and samples in serological and molecular tests were interpreted based on their collection site (urban or rural areas). Infection prevalence was calculated following the method proposed by Noordhuizen et al.^51^ and Bennett et al.,^52^ using the following formula:

$$\mathrm{Prevalence}=\frac{n}{N}$$

where *n* is the number of seropositive/positive animals in MAT or PCR, and *N* is the total number of tested animals.

Finally, associations between geographic area (urban vs. rural) and *Leptospira* detection, based on seroreactivity (MAT) and PCR positivity, were assessed using frequency distribution, and comparisons were made using Fisher's exact test at a 5% significance level.

## RESULTS

A total of 16 (64%) cane toads were collected from urban areas, and nine (36%) from rural environments (Table 3). Of the 25 toads, six (24%) tested seroreactive by MAT, with antibody titres ranging from 1:100 to 1:400. No observable clinical signs of leptospirosis, such as cutaneous lesions (ulcers, erythema or areas of skin necrosis), oedema, changes in skin colouration or dehydration evidenced by dry or wrinkled skin, were detected in any of the toads.

**TABLE 3:** Serogroup, microscopic agglutination testing (MAT) titre and polymerase chain reaction (PCR) results (kidney tissue) of cane toads captured in urban and rural areas of Patos municipality, Paraíba State, Brazil.

Sample	Area	MAT result	Serogroup	Titre	PCR—kidney tissue
AH1	Urban zone	Seronegative	None	0	Negative
AH2	Urban zone	Seronegative	None	0	Positive
AH3	Urban zone	Seronegative	None	0	Positive
AH4	Urban zone	Seronegative	None	0	Positive
AH5	Urban zone	Seropositive	Pomona	200	Positive
AH6	Urban zone	Seropositive	Icterohaemorrhagiae	400	Positive
AH7	Urban zone	Seronegative	None	0	Negative
AH8	Rural zone	Seropositive	Australis	200	Positive
AH9	Rural zone	Seronegative	None	0	Positive
AH10	Rural zone	Seronegative	None	0	Negative
AH11	Urban zone	Seronegative	None	0	Positive
AH12	Urban zone	Seronegative	None	0	Negative
AH13	Rural zone	Seronegative	None	0	Negative
AH14	Rural zone	Seronegative	None	0	Negative
AH15	Rural zone	Seropositive	Australis	100	Negative
AH16	Urban zone	Seronegative	None	0	Negative
AH17	Urban zone	Seronegative	None	0	Positive
AH18	Urban zone	Seronegative	None	0	Negative
AH19	Urban zone	Seronegative	None	0	Negative
AH20	Urban zone	Seropositive	Icterohaemorrhagiae	100	Negative
AH21	Urban zone	Seronegative	None	0	Negative
AH22	Urban zone	Seronegative	None	0	Positive
AH23	Rural zone	Seronegative	None	0	Negative
AH24	Rural zone	Seropositive	Australis	100	Negative
AH25	Rural zone	Seronegative	None	0	Positive

Seroreactivity was most frequently observed against the Australis and Icterohaemorrhagiae serogroups, with antibody titres suggesting prior exposure in 12% and 8% of toads from urban and rural areas, respectively. Additionally, one toad from the urban area exhibited seroreactivity to the Pomona serogroup (4%). However, it is important to note that the infecting serovar cannot be definitively determined based on MAT results.

Molecular analysis targeting the *LipL32* gene of *Leptospira* spp. confirmed infection in 11 toads (44%) (Table 4), including eight from urban areas and three from rural zones. Among the six toads that tested positive for anti‐*Leptospira* antibodies by MAT, only three also presented *Leptospira* DNA in kidney tissue, as detected by PCR.

**TABLE 4:** Summary of the microscopic agglutination test (MAT) and polymerase chain reaction (PCR) results for the 25 cane toads (*Rhinella jimi*) assessed in this study.

MAT	PCR performed on kidney tissue		Total (%)
	Positive	Negative	
Seropositive	3	3	6 (24%)
Seronegative	8	11	19 (76%)
Total (%)	11 (44%)	14 (56%)	25 (100%)

Using MAT, the prevalence of anti‐*Leptospira* agglutinins detected was 24%, while the PCR technique revealed a positivity prevalence of 44%.

Table 5 presents the seroprevalence (proportion of *R. jimi* toads seropositive by MAT) and infection prevalence (proportion of toads positive by PCR) according to geographic area (urban vs. rural). Fisher's exact test showed no statistically significant difference in seroprevalence between rural (33.3%) and urban toads (18.7%; *p* = 0.63). Similarly, PCR‐based infection prevalence did not differ significantly between urban (50%) and rural toads (33.3%; *p* = 0.68).

**TABLE 5:** Association between the area of ​​origin of the cane toads (*Rhinella jimi*) and the results obtained in the microscopic agglutination test (MAT) and polymerase chain reaction (PCR).

Diagnostic test	Result category	Urban (*n* = 16)	Rural (*n* = 9)	*p*‐Value
MAT	Seropositive	3 (18.7%)	3 (33.3%)	0.63
	Seronegative	13 (81.3%)	6 (66.7%)	
PCR	Positive	8 (50.0%)	3 (33.3%)	0.68
	Negative	8 (50.0%)	6 (66.7%)	

*Note*: Data were analysed using Fisher's exact test.

## DISCUSSION

This study's findings raise important questions regarding the broad host range of *Leptospira* and the potential role of non‐conventional hosts in its maintenance and transmission in highly biodiverse tropical regions.^3^ Notably, the detection of 11 PCR‐positive toads (44%) represents previously unreported bacterial colonisation in this species within the region, suggesting new potential reservoirs of *Leptospira*.

Two complementary scenarios emerge from the results. MAT‐seronegative but PCR‐positive samples indicate that the toads were renal carriers of *Leptospira*, despite exhibiting low or undetectable levels of anti‐*Leptospira* agglutinins in serum. Conversely, MAT‐seropositive but PCR‐negative samples most likely reflect individuals who experienced a prior *Leptospira* infection and subsequently recovered, as circulating antibodies indicate past exposure only and not active renal colonisation. Other possible explanations include antibody production without renal colonisation, cross‐reactivity with other serovars, or the presence of non‐pathogenic strains not detected by the LipL32 gene‐based assay, none of which would pose a zoonotic risk.

The high proportion of PCR‐positive animals, including seronegative individuals, and the low MAT reactivity titres, starting from 1:100, suggest that the anurans may have been in a chronic carrier phase, in which antibody levels gradually decline to values undetectable by serology; or they could have been in an early stage of infection, prior to the development of detectable agglutinins. Additionally, it is also possible that some individuals do not mount a strong antibody response, resulting in low or absent MAT seroreactivity despite renal colonisation.^53^, ^54^ The lack of data on antibody titration and titre maintenance in wild animals hinders the determination of an appropriate cutoff point for these species, considering the influence of regional environmental factors.^54^

Although MAT identifies the serogroups with which the host serum reacts, it does not confirm the specific *Leptospira* serovar responsible for the infection.^55^ Therefore, seropositivity in toads should be interpreted with caution, as it does not necessarily confirm current or prior infection by the Icterohaemorrhagiae, Pomona or Australis serogroups, given known cross‐reactivity. Reactions to the Icterohaemorrhagiae serogroup, commonly found in rodents, the primary reservoir in Brazil, and responsible for most human leptospirosis cases, indicate prior exposure to *Leptospira* and highlight potential environmental risk.^56^, ^57^

Similarly, reactivity to the Pomona serogroup may reflect environmental exposure in areas with synanthropic species such as opossums, rodents and domestic dogs and cats.^58^ The Pomona serogroup, although frequently associated with livestock, particularly pigs, has also been widely detected in wildlife, including mesocarnivores and other terrestrial mammals across the Americas.^59^ In Brazil, it shows notable prevalence in the Northeastern region, while in the United States, it has been reported in Texas, Oregon, California, Iowa and Hawaii.^6^, ^60^, ^61^

No significant difference in seropositivity was observed between anurans from urban and rural areas, indicating that exposure to *Leptospira* occurs in both environments. Most seropositive animals exhibited low titres, possibly due to post‐infection decline, weak immune response or recent exposure to the pathogen.^62^ However, Rodrigues et al.^63^ reported that even low titres in rattlesnakes are significant, positioning these animals as potential contributors to environmental dissemination despite the absence of clinical signs. A similar inference can be applied to amphibians, given the limited data on seroprevalence and immunological responses during *Leptospira* infection.

PCR and MAT play complementary roles: PCR detects leptospiral DNA even in non‐viable organisms,^64^, ^65^ while MAT provides information on serological exposure.^66^ Their combined use supports epidemiological investigations.^39^, ^67^, ^68^ In domestic animals from the semi‐arid region, studies have typically used a cutoff of 1:100 for MAT.^39^, ^69^, ^70^, ^71^ However, Suárez and Parra^72^ observed that a 1:50 dilution reduced false negatives in cattle in Colombia. In wild animals, cutoff points also vary: Pimentel et al.,^73^ Paixão et al.,^74^ Brasil et al.^75^ and Horta et al.^76^ used 1:100, while Silva et al.^77^ considered animals with titres of 1:40 or more as reactive. These differences highlight the lack of consensus and underscore that MAT seropositivity reflects exposure to *Leptospira* rather than definitive infection or clinical leptospirosis, emphasising that interpretation should consider both titre magnitude and epidemiological context.

Additionally, in human populations, leptospirosis is widely recognised as a major public health concern in urban areas, with Brazil exhibiting the highest prevalence in Latin America.^78^ The disease primarily affects tropical regions, where approximately 73% of cases are reported, particularly in Southeast Asia, East Sub‐Saharan Africa, the Caribbean and Oceania. Moreover, it is most frequently observed in rural agricultural communities and in vulnerable urban and peri‐urban areas, disproportionately affecting young adult males.^79^

No significant difference in infection or exposure was observed between toads from urban and rural sites. The limited sample size may explain the absence of statistically significant differences along the urban‒rural gradient. Alternatively, this pattern may indicate that the transmission risk is relatively uniform across the landscape, without marked spatial variation.

In conclusion, the epidemiological cycle of *Leptospira* is shaped by regional factors, requiring diverse approaches to elucidate reservoirs and hosts. Although the study's sample size is ethically justified, it constrains the generalisability of the findings. Further research with broader sampling, culture isolation and phylogenetic analyses is essential to clarify interspecies epidemiological connections in leptospirosis.

## CONCLUSION

The presence of anti‐*Leptospira* spp. antibodies and leptospiral DNA was confirmed in cane toads (*R. jimi*) captured in urban and rural areas of Paraíba state, Brazil, a region characterised by a hot and dry semi‐arid climate with scarce and concentrated rainfall. These findings suggest that this synanthropic amphibian species may act as a ‘non‐conventional’ host in the epidemiological cycle of leptospirosis, elucidating the potential transmission risk that toads may pose to people and domestic animals.

The serological and molecular findings indicate contact of these amphibians with serogroups related to *L. interrogans*, the pathogenic species most frequently associated with leptospirosis cases in mammals. Further research is warranted to fully elucidate the effective role exerted by the species *R. jimi* in the maintenance and propagation of this zoonosis, as well as to characterise its immunological response following infection with *Leptospira* spp.

## AUTHOR CONTRIBUTIONS

*Writing—review and editing, writing—original draft, methodology, investigation, formal analysis and conceptualisation*: Karla N. de Souza Rocha. *Methodology, formal analysis, data curation and conceptualisation*: Aline de Lima Peixoto, Lucila S. de Medeiros Pereira and Debora Ferreira Cardoso. *Formal analysis, data curation and software*: Juliana P.F. de Oliveira and Maria L.C. Rodrigues Silva. *Writing—review and editing, methodology and supervision*: Sérgio Santos de Azevedo. *Writing—review and editing, supervision, methodology and conceptualisation*: Clebert J. Alves. *Writing—review and editing, and supervision*: Mozar Alves Fonseca. *Writing—review and editing, supervision, resources, methodology and conceptualisation*: Severino S. dos Santos Higino.

## CONFLICT OF INTEREST STATEMENT

The authors declare they have no conflicts of interest.

## FUNDING INFORMATION

The authors received no specific funding for this work.

## ETHICS STATEMENT

This research was approved by the Animal Use Ethics Committee of the Federal University of Campina Grande (FUCG) under opinion no. 089/2019, and by the Brazilian Institute of Environment and Renewable Natural Resources (BIERNR)/Chico Mendes Institute for Biodiversity Conservation (ICMBio—Brazil) under protocol no. 70478‐1.

## Data Availability

The registration data of the animals and the results of the serological and molecular analyses found in the study are available from the author upon reasonable request.
